# Structural Brain Correlates of Attention Dysfunction in Lewy Body Dementias and Alzheimer’s Disease

**DOI:** 10.3389/fnagi.2018.00347

**Published:** 2018-10-30

**Authors:** Ruth A. Cromarty, Julia Schumacher, Sara Graziadio, Peter Gallagher, Alison Killen, Michael J. Firbank, Andrew Blamire, Marcus Kaiser, Alan J. Thomas, John T. O’Brien, Luis R. Peraza, John-Paul Taylor

**Affiliations:** ^1^Institute of Neuroscience, Campus for Ageing and Vitality, Newcastle University, Newcastle upon Tyne, United Kingdom; ^2^NIHR In Vitro Diagnostics Co-operative, Newcastle upon Tyne Hospitals NHS Foundation Trust, Medical School, Newcastle University, Newcastle upon Tyne, United Kingdom; ^3^Institute of Neuroscience, Newcastle University, Newcastle upon Tyne, United Kingdom; ^4^Newcastle Magnetic Resonance Centre, Campus for Ageing and Vitality, Newcastle University, Newcastle upon Tyne, United Kingdom; ^5^Interdisciplinary Computing and Complex BioSystems (ICOS) Research Group, School of Computing, Newcastle University, Newcastle upon Tyne, United Kingdom; ^6^Department of Psychiatry, University of Cambridge School of Medicine, Cambridge, United Kingdom

**Keywords:** attention network test, reaction times, orienting, alerting, executive conflict, voxel-based morphometry, dementia with Lewy bodies, Parkinson’s disease dementia

## Abstract

Lewy body dementia (LBD) and Alzheimer’s disease (AD) are common forms of dementia that have different clinical profiles but are both commonly associated with attentional deficits. The aim of this study was to investigate efficiency of different attentional systems in LBD and AD and its association with brain structural abnormalities. We studied reaction time (RT) data from 45 LBD, 31 AD patients and 22 healthy controls (HCs) using the Attention Network Test (ANT) to assess the efficiency of three different attentional systems: alerting, orienting and executive conflict. Voxel-based morphometry (VBM) was used to investigate relations between different attention components and cortical volume. Both dementia groups showed slower overall RTs than controls, with additional slowing in LBD relative to AD. There was a significant alerting effect in controls which was absent in the dementia groups, the executive conflict effect was greater in both dementia groups compared to controls, but the orienting effect did not differ between groups. Mean RT in AD was negatively correlated with occipital gray matter (GM) volume and in LBD orienting efficiency was negatively related to occipital white matter (WM) volume. Given that previous studies in less impaired patients suggest a maintenance of the alerting effect, the absent alerting effect in our study suggests a loss of alerting efficiency with dementia progression. While orienting was largely preserved, it might be related to occipital structural abnormalities in LBD. Executive function was markedly impaired in both dementia groups, however, the absence of relations to brain volume suggests that it might be more related to functional rather than macrostructural pathophysiological changes.

## Introduction

Lewy body dementia (LBD) is the second most common form of neurodegenerative dementia in older age after Alzheimer’s disease (AD) and includes dementia with Lewy bodies (DLB) and Parkinson’s disease with dementia (PDD; McKeith, 2007). Core clinical features associated with LBD include fluctuations in cognition and attention, Parkinsonism and complex visual hallucinations (McKeith et al., 2005, 2017). DLB and PDD are differentiated from each other according to the onset of dementia relative to Parkinsonism; DLB is diagnosed if dementia onset is less than one year after Parkinsonism onset, PDD if more than one year elapses prior to dementia onset (McKeith et al., 2005, 2017; Aarsland et al., 2009).

Previous investigations using attention tasks found that LBD patients show slower reaction times (RTs) and higher intra-individual variability than AD patients (Ballard et al., 2001; Bradshaw et al., 2004), and that this difference is accentuated when the task involves an executive or spatial aspect (Bradshaw et al., 2004).

The cognitive function of attention can be modeled as three anatomically distinct, but functionally inter-related systems: alerting, orienting and executive control or conflict (Posner and Petersen, 1990). The efficiency of these three attention systems is commonly assessed using the Attention Network Test (ANT, Fan et al., 2002) which combines elements of the Posner cueing paradigm (Posner and Petersen, 1990) and the Eriksen flanker task (Eriksen and Eriksen, 1974) to test all three components in a single session. The significance and size of the alerting, orienting and executive conflict effects is measured by differences in RT performance between different cue and target conditions. First, the alerting effect is assessed as the benefit of a simple warning cue on subsequent RT performance. The size of this effect is therefore an indicator for the ability to achieve and maintain a vigilant or alert state (Posner and Petersen, 1990). Second, the orienting effect is measured by the difference in RT when presenting stimuli in a previously cued location in space compared to an uncued location. Its size therefore indicates how efficiently a participant can select information from sensory input (Fan et al., 2005). Finally, the size of the executive conflict effect is tested using a flanker task, i.e., by measuring the effect of distracting targets on RT performance (Eriksen and Eriksen, 1974). An increase in the executive conflict effect thus indicates an impairment in resolving conflict amongst responses.

To date, very little research has been conducted to investigate how the efficiency of these different attentional systems is affected by dementia and the findings of previous studies are inconsistent (Fernandez-Duque and Black, 2006; Fuentes et al., 2010). The first objective of this study was therefore to determine the extent to which the efficiency of the attentional systems is differentially affected in LBD patients relative to AD patients and age-matched healthy participants. The alerting component is purported to be modulated by noradrenergic projections from the locus coeruleus (Coull et al., 2001; Raz, 2004). Given that LBD patients have been found to have a paucity of noradrenergic neurons in the locus coeruleus (Szot et al., 2006), our first hypothesis was therefore that we would find reduced alerting efficiency in LBD patients. Secondly, as the orienting component has been suggested to be modulated by the basal forebrain cholinergic system (Fan et al., 2005) which is more markedly affected in LBD patients than in AD (Ballard et al., 2013), we hypothesized that LBD patients would also exhibit differentially reduced orienting efficiency compared to AD and controls. Thirdly, based on previous literature we hypothesized that AD patients would exhibit reduced executive conflict efficiency relative to healthy controls (HCs). Given that deficits in executive functioning are also a common feature of LBD, it was hypothesized that the LBD group would also exhibit reduced executive conflict processing efficiency. Furthermore, executive control has been shown to be modulated by the dopaminergic and cholinergic systems (Noudoost and Moore, 2011) which are more affected in LBD compared to AD. Thus, we hypothesized to find a difference in executive conflict efficiency between the dementia groups with a greater impairment in LBD compared to AD.

Previous studies have also investigated how inter-individual differences in the efficiency of the attentional systems as measured by the ANT were related to differences in brain structure in healthy participants (Westlye et al., 2011; Hao et al., 2015) and amnestic mild cognitive impairment (aMCI, Borsa et al., 2016). However, to our knowledge no-one has yet reported how the efficiency of these attentional systems is related to structural alterations in more severe neurodegenerative disease. Our aim was therefore to study macrostructural neural correlates of attentional dysfunction in AD and LBD using a voxel-based morphometry (VBM) analysis. In a previous investigation, Borsa et al. (2016) found an association between impairment in the executive conflict component in aMCI patients and decreased gray matter (GM) volume in the anterior cingulate cortex (ACC). Given that aMCI is associated with a high conversion rate to AD (Petersen, 2004), we hypothesized that there might be a similar or possibly even stronger relationship between conflict monitoring and brain volume in the ACC in our AD group. However, given the general paucity of previous research in this area, our analysis of brain structural correlates of the attentional systems was conducted in a more exploratory manner, using a whole-brain approach rather than restricting the analysis to *a priori* defined regions.

Finally, we were interested in studying how the different ANT effects were influenced by the clinical LBD symptoms. We hypothesized that we might find correlations between the size of the ANT effects and cognitive fluctuation severity as cognitive fluctuations have been found to be related to other RT measures in DLB (Walker et al., 2000; Bradshaw et al., 2006).

## Materials and Methods

### Participants

The study involved 105 participants who were over 60 years of age. Fifty were diagnosed with probable LBD, 33 with probable AD and 22 were age-matched HC. The LBD group comprised patients diagnosed with DLB (*N* = 26) and PDD (*N* = 24).

Patients were recruited from the local community-dwelling population who had been referred to old age psychiatry and neurology services. Dementia was diagnosed independently by two experienced old age psychiatrists in alignment with consensus criteria for probable DLB (McKeith et al., 2005, 2017), PDD (Emre et al., 2007) and AD (McKhann et al., 2011). HC were recruited from friends/acquaintances of the patients, with an Mini Mental State Examination (MMSE) ≥28 and no history of psychiatric or neurological illness. This study was carried out in accordance with the recommendations of Newcastle Ethics committee with written informed consent from all subjects. All participants gave written informed consent in accordance with the Declaration of Helsinki. The ethics statement is a bit repetitive. Maybe the last sentence could be removed (‘The protocol was approved by the Newcastle Ethics committee.’) as it repeats information from the first sentence.

It was decided *a priori* to combine DLB and PDD patients into one LBD group as previous studies have shown similar attentional and executive impairment in DLB and PDD (Ballard et al., 2002; Mondon et al., 2007; Firbank et al., 2016) and similar patterns of brain structural (Burton et al., 2004) and functional (Peraza et al., 2015) alterations.

The datasets for this manuscript are not publicly available because of limitations due to participant consent. Requests to access the datasets should be directed to Dr John-Paul Taylor (john-paul.taylor@ncl.ac.uk).

### Modified Attention Network Test

**Figure 1:**
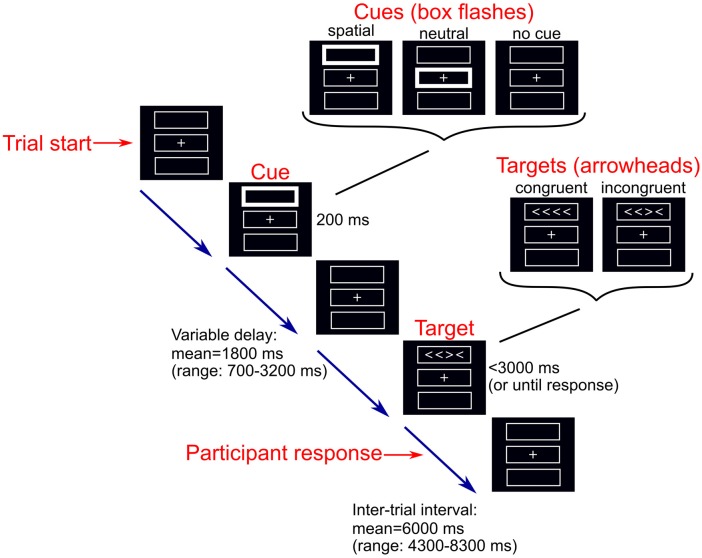
Depiction of a single trials of the modified Attention Network Test (ANT).

We used a modified version of the ANT that can be performed without difficulty by older adults and dementia patients (Firbank et al., 2016). The computerized task was programmed using the Cogent MATLAB toolbox^1^ (Figure 1). Participants completed between 3 and 14 runs of the task (median = 8) of 36 trials each. Throughout the task a central fixation cross and three boxes were present on a screen. During each trial, one of three possible cues (no, neutral, or spatial cue) was presented for 200 ms. During the presentation of a neutral cue, the central box flashed. In the spatial cue condition, one of the boxes either above or below the central fixation flashed (indicating the box in which a subsequent target would appear). In the no cue condition, the boxes remained unchanged. Following the disappearance of the cue, a target comprising four arrowheads in a row was presented in either the box above or below the central box. The target stimuli were either congruent or incongruent; congruent targets comprised arrowheads which were all pointing in the same direction (left or right), whereas for incongruent target stimuli one arrowhead was pointing in the opposite direction to the other arrowheads. The target remained on screen until the participant responded, by squeezing a right or left hand air pressure bulb to indicate the direction in which the majority of the arrowheads were facing, or until 3,000 ms had elapsed. During each run, the six trial types were presented in a predetermined counterbalanced order; each cue appeared 12 times, there were 18 congruent trials and 18 incongruent trials. All trials from runs with less than 2/3 correct responses were excluded from further analysis as performance below this was not different from chance (Firbank et al., 2016).

### Analysis of ANT Effects

Mean RTs and standard deviations for each cue and target condition were calculated in Matlab (R2015b), using only the trials in which the participants gave correct responses in alignment with previous studies. The ANT effects were calculated as defined by Fan et al. (2002):

$$\begin{array}{c} \text{Alerting effect = no cue}{\text{ trials}}_{\text{mean RT}}-\text{neutral cue}{\text{ trials}}_{\text{mean RT}}\\ \text{Orenting effect = no cue}{\text{ trials}}_{\text{mean RT}}-\text{spatial cue}{\text{ trials}}_{\text{mean RT}}\\ \text{Executive conflict effect = incongruent target}{\text{ trials}}_{\text{mean RT}}-\text{congruent target}{\text{ trials}}_{\text{mean RT}} \end{array}$$

The alerting effect is therefore a measure of the extent to which response speed is facilitated by the presence of a warning, indicating that a response is imminently required. The orienting effect is the extent to which responses are further facilitated when the actual spatial location of the oncoming target is cued, rather than a simple warning that a response is imminent. Finally, the executive conflict effect pools all types of cued condition and examines the impairing/interfering effect of having conflicting information regarding the target stimuli (in terms of the direction in which each of the arrowheads are pointing), compared to the facilitative effect of having target stimuli which are all pointing in the same direction.

To calculate the alerting and orienting effects, the mean RTs were averaged across the congruent and incongruent target trials. Similarly, the mean RTs for the target trials used in the executive conflict effect were calculated by averaging across the cue conditions. Error rates were also determined for each task condition by dividing the number of incorrect and missed response trials by the number of recorded trials for each participant.

### Magnetic Resonance Imaging and Analysis

Structural MR images were acquired with a 3 T Philips Intera Achieva scanner with a magnetization prepared rapid gradient echo (MPRAGE) sequence, sagittal acquisition, echo time 4.6 ms, repetition time 8.3 ms, inversion time 1,250 ms, flip angle = 8°, SENSE factor = 2 and in-plane field of view 240 × 240 mm^2^ with slice thickness 1.0 mm, yielding a voxel size of 1.0 × 1.0 × 1.0 mm^3^.

A VBM analysis was performed in SPM12 (Statistical Parametric Mapping^2^), to assess voxelwise correlations between the ANT results and mean RT and GM and white matter (WM) volume. Images were segmented into GM, WM and cerebrospinal fluid. The segmented GM and WM images were co-registered and normalized to MNI space using SPM’s DARTEL algorithm (Ashburner, 2007) and modulated. Finally, images were smoothed with an 8 mm full width at half maximum Gaussian kernel.

### Statistics

For the mean RT data for each task condition, a repeated measures (cue × target) ANOVA was conducted with a between-subject factor of group (HC, AD and LBD). Subsequently, separate repeated measures (cue × target) ANOVAs were conducted for each group; *post hoc* pairwise comparisons were used to calculate RT differences between the cue and target conditions, thus determining whether the ANT effects were significant. The magnitude of the ANT effects was compared between groups using univariate ANOVAs; the dependent variable being the ANT effect with group as a fixed factor. For each of the ANOVA analyses discussed, Mauchly’s sphericity test was used and *F*-values adjusted accordingly. *Post hoc* pairwise comparisons were performed using Bonferroni correction for family wise errors. The same analysis was repeated for the error rates. Additionally, to control for the effect of overall processing speed and to test whether between-group differences in overall processing speed influenced the analyses of the ANT effects, we repeated all analyses using normalized RT; for each participant, the mean RT for each condition was divided by the participant’s overall mean RT in alignment with previous studies (Faust and Balota, 1997; Fernandez-Duque and Black, 2006).

Correlation analyses (using Pearson’s correlation) were conducted to investigate associations between the behavioral data (overall mean RT and the ANT effects) and clinical variables in the dementia groups. Clinical variables investigated included: measures of global cognition in AD and LBD (Cambridge Cognitive Examination (CAMCOG) and MMSE total scores), measures of cognitive fluctuations in LBD (Mayo fluctuations and Clinician Assessment of Fluctuation (CAF) total and subscores), Unified Parkinson’s Disease Rating Scale (UPDRS) motor subscale in LBD and the NPI visual hallucinations score in LBD. *P*-values were FDR-corrected for multiple comparisons.

Correlations between the ANT behavioral data and GM and WM volume were assessed using a general linear model (GLM) in SPM12. The GLM combined all three ANT effects (alerting, orienting and executive conflict) as variables of interest in one design matrix and a separate model was used for mean RT. Covariates of no interest for age, gender, total intracranial volume and UPDRS motor scores (in LBD) were included. An explicit mask was estimated (Ridgway et al., 2009) to restrict the statistical analysis to voxels which represented GM and WM, respectively. Significant results are reported at a voxel-level *p*-value < 0.001. Additionally, the minimum cluster size for a multiple comparison corrected threshold of *p* < 0.05 was determined by Monte Carlo simulations using the REST software^3^.

## Results

### Demographics

Two AD, one DLB and four PDD patients were excluded from the study because they did not meet the minimum performance criteria. This resulted in 31 AD, 45 LBD (25 DLB and 20 PDD) and 22 HC participants for further analysis (Table 1). All groups were matched for age and gender. LBD patients were slightly less impaired in terms of overall cognition (MMSE and CAMCOG) and had shorter duration of cognitive symptoms compared to AD patients. The proportion of patients taking cholinesterase inhibitors did not differ between the dementia groups, whereas more LBD patients were taking dopaminergic medication compared to AD. As expected, LBD patients were more impaired in terms of the core LBD symptoms of Parkinsonism, cognitive fluctuations and visual hallucinations compared to the AD group.

**Table 1:** Demographics and clinical information; mean (standard deviation).

	HC (*n* = 22)	AD (*n* = 31)	LBD (*n* = 45)	Between-group differences
Male: female	15:7	24:7	38:7	*χ*^2^ = 2.36, *p* = 0.31^a^
Age	75.9 (5.4)	77.1 (7.9)	74.5 (6.3)	*F*_(2,95)_ = 1.41, *p* = 0.25^b^
AChEI	na	28	39	*χ*^2^ = 0.24, *p* = 0.63^c^
Dopaminergic medication	na	0	33	*χ*^2^ = 40.2, *p* < 0.001^c^
Duration	na	3.9 (2.1)	3.1 (2.1)	*U* = 509, *p* = 0.043^d^
MMSE	29.2 (0.9)	21.1 (3.7)	23.3 (3.8)	*t*_(74)_ = 2.5, *p* = 0.01^e^
CAMCOG	96.7 (3.7)	68.8 (13.3)	75.9 (12.6)	*t*_(74)_ = 2.4, *p* = 0.01^e^
UPDRS	1.1 (1.4)	2.4 (2.2)	20.5 (9.3)	*t*_(74)_ = 10.6, *p* < 0.001^e^
CAF total	na	0.8 (1.7)^f^	5.1 (4.5)^g^	*t*_(71)_ = 4.9, *p* < 0.001^e^
Mayo total	na	9.1 (4.1)^f^	13.5 (5.8)^g^	*t*_(71)_ = 3.6, *p* = 0.001^e^
Mayo cogn	na	1.8 (1.8)^f^	2.8 (1.9)^g^	*t*_(71)_ = 2.4, *p* = 0.02^e^
NPI total	na	6.9 (6.2)^f^	13.4 (9.7)	*t*_(73)_ = 3.3, *p* = 0.002^e^
NPI hall	na	0.03 (0.2)^f^	1.8 (2.0)	*t*_(73)_ = 4.8, *p* < 0.001^e^

*AChEI, number of patients taking acetylcholinesterase inhibitors; AD, Alzheimer’s disease; CAF total, Clinical Assessment of Fluctuations total score; CAMCOG, Cambridge Cognitive Examination; Duration, duration of cognitive symptoms in years; HC, healthy controls; LBD, Lewy body dementia; Mayo Fluctuations, Mayo Fluctuations cognitive subscale; MMSE, Mini Mental State Examination; na, not applicable; Dopaminergic medication, number of patients taking dopaminergic medication; UPDRS, Unified Parkinson’s Disease Rating Scale III; NPI, Neuropsychiatric Inventory; NPI hall, NPI hallucination subscore. ^a^Chi-square test HC, AD, LBD; ^b^One-way ANOVA HC, AD, LBD; ^c^Chi-square test AD, LBD; ^d^Mann Whitney U test AD, LBD; ^e^Student’s *t*-test AD, LBD; ^f^*N* = 30, ^g^*N* = 43*.

To ensure that differences between the dementia groups in terms of behavioral data were not due to the differences in MMSE, we repeated all analyses reported below with matched dementia groups (see “Analysis of Matched Dementia Subgroups” section of the Supplementary Table S11). Results from this additional analysis were broadly similar to the results from the whole group (Supplementary Table S12).

There were no significant differences between DLB and PDD subgroups in terms of age, overall cognition and dementia duration, while PDD patients had more severe Parkinsonism, psychiatric symptoms and cognitive fluctuations than DLB patients (Supplementary Table S1). To ensure that it was appropriate to combine DLB and PDD patients into one LBD group, mean RT, error rates and the different ANT effects were compared between the two groups showing no significant differences (Supplementary Table S2).

### Reaction Time Analysis

The number of recorded trials per participant did not differ between the groups (mean (SD) HC: 301.1 (17.7), AD: 290.3 (41.5), LBD: 304.8 (37.3); univariate ANOVA, *F*_(2,95)_ = 1.56, *p* = 0.22). Overall, the percentage of correct trials was higher in the control group than in AD and LBD but did not significantly differ between the dementia groups (Supplementary Tables S3, S4A).

#### Overall Mean Reaction Time

**Figure 2:**
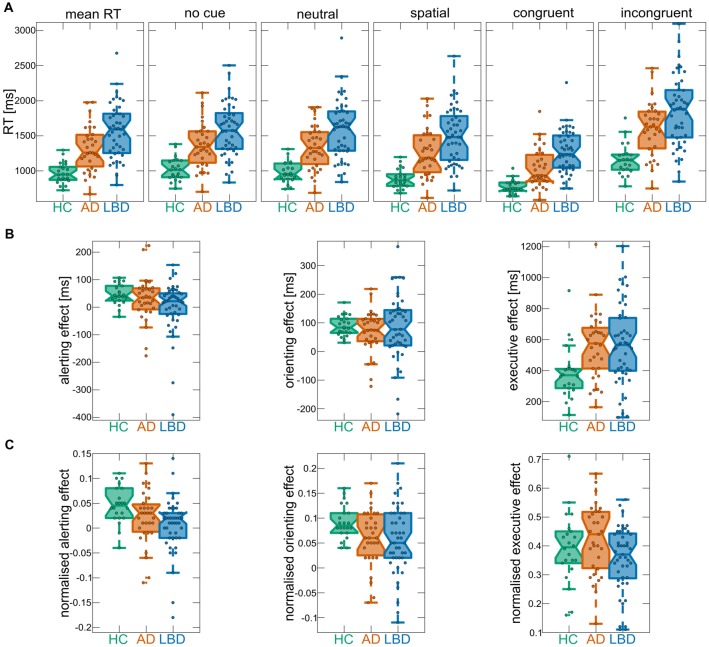
**(A)** Mean reaction times (RTs) overall and for the different cue and target conditions of the ANT within each group. **(B)** ANT effects from raw RTs. **(C)** ANT effects from normalized RTs. In each boxplot the central line corresponds to the sample median, the upper and lower border of the box represent the 25th and 75th percentile, respectively, and the length of the whiskers is 1.5 times the interquartile range. Corresponding results from statistical comparisons between the three groups are presented in Table 3. AD, Alzheimer’s disease; HCs, healthy controls; LBD, Lewy body dementia.

**Figure 3:**
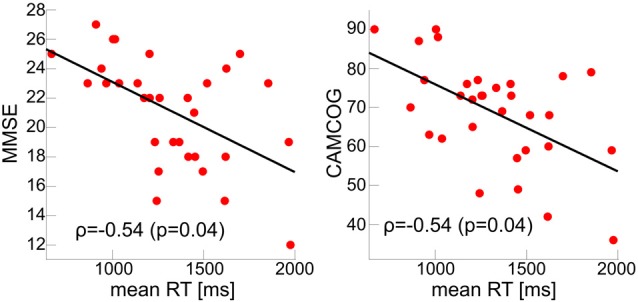
Correlations between global cognition and mean RT with FDR-corrected *p*-values < 0.05 in the AD group. MMSE, Mini Mental State Examination; CAMCOG, Cambridge Cognitive Examination.

Mean RTs were normally distributed within each group (Kolmogorov-Smirnov test, all *p* > 0.05). There was a main effect of group across all cue and target conditions; control participants had faster overall mean RT relative to AD and LBD patients, and LBD mean RT was slower than in AD (Tables 2, 3A, Figure 2). Mean RT was significantly negatively correlated with overall cognition (MMSE and CAMCOG) in AD (Figure 3).

**Table 2:** Mean reaction time (RT) from correct trials (ms) for each task condition (cue × target), for the controls, AD and LBD patients.

		HC (*n* = 22)	AD (*n* = 31)	LBD (*n* = 45)
**(A) Mean reaction time (ms)**				
All trials		964.6 (147.5)	1319.8 (322.6)	1558.3 (391.0)
No Cue	Overall	1025.1 (162.6)	1363.2 (325.3)	1587.0 (377.4)
	Congruent	806.7 (110.2)	1059.8 (270.2)	1280.4 (288.4)
	Incongruent	1243.4 (241.4)	1666.6 (402.4)	1893.6 (491.9)
Neutral	Overall	978.9 (147.3)	1334.1 (309.5)	1585.9 (413.0)
	Congruent	795.7 (98.8)	1072.0 (267.7)	1293.7 (302.6)
	Incongruent	1162.0 (222.4)	1596.1 (383.6)	1878.2 (553.6)
Spatial	Overall	900.00 (136.4)	1262.0 (342.9)	1502.0 (395.8)
	Congruent	710.5 (89.5)	989.1 (314.1)	1245.7 (355.7)
	Incongruent	1069.5 (203.5)	1534.9 (411.0)	1758.4 (478.9)
Congruent	Overall	771.0 (97.6)	1040.3 (278.0)	1273.3 (302.8)
Incongruent	Overall	1158.3 (220.8)	1599.2 (393.0)	1843.4 (496.4)
**(B) ANT effects (ms)**				
Alerting		46.2 (37.0)*	29.14 (80.84)	1.1 (93.7)
Orienting		88.9 (35.0)*	72.07 (74.62)*	83.9 (112.9)*
Executive conflict		387.4 (171.9)*	558.88 (217.13)*	570.1 (254.2)*

Standard deviations are presented in brackets. AD, Alzheimer’s disease; HC, healthy controls; LBD, Lewy body dementia; *Significant attention network test (ANT) effect, *p*-value < 0.05 for normalized RTs.

**Table 3:** Results from statistical tests for raw and normalized RTs.

		Effect significance, raw RT	Effect significance, normalized RT
**Main effects**			
**(A)** Group		*F*_(2,95)_ = 24.19, *p* < 0.001	
	HC-AD	(−578.9, −131.4), *p* = 0.001	
*Post hoc*	HC-LBD	(−802.5, −384.9), *p* < 0.001	
	AD-LBD	(−425.9, −51.2), *p* = 0.008	
**(B)** Cue		*F*_(2,190)_ = 73.97, *p* < 0.001	*F*_(2,190)_ = 131.81, *p* < 0.001
*Post hoc*	Alerting	(4.9, 46.1), *p* = 0.01	(0.011, 0.038), *p* < 0.001
	Orienting	(58.7, 104.5), *p* < 0.001	(0.055, 0.085), *p* < 0.001
**(C)** Target		*F*_(1,95)_ = 448.04, *p* < 0.001	*F*_(1,95)_ = 981.64, *p* < 0.001
**Interactions**			
**(D)** Cue × Group		*F*_(4,190)_ = 1.64, *p* = 0.17	*F*_(4,190)_ = 6.97, *p* < 0.001
HC Cue		*F*_(2,42)_ = 167.0, *p* < 0.001
	Alerting		(0.026, 0.068), *p* < 0.001
	Orienting		(0.073, 0.111), *p* < 0.001
AD Cue		*F*_(2,60)_ = 33.33, *p* < 0.001
	Alerting		(−0.003, 0.047), *p* = 0.10
	Orienting		(0.037, 0.089), *p* < 0.001
LBD Cue		*F*_(2,88)_ = 24.89, *p* < 0.001
	Alerting		(−0.015, 0.026), *p* = 1.0
	Orienting		(0.03, 0.080), *p* < 0.001
**(E)** Target × Group		*F*_(2,95)_ = 5.30, *p* = 0.007	*F*_(2,95)_ = 3.01, *p* = 0.054
HC	Executive	*F*_(1,21)_ = 111.68, *p* < 0.001	*F*_(1,21)_ = 227.05, *p* < 0.001
AD	Executive	*F*_(1,30)_ = 205.38, *p* < 0.001	*F*_(1,30)_ = 370.25, *p* < 0.001
LBD	Executive	*F*_(1,44)_ = 226.33, *p* < 0.001	*F*_(1,44)_ = 443.46, *p* < 0.001
**(F)** Cue × target		*F*_(1.7,157.1)_ = 6.51, *p* = 0.002	*F*_(1.9,177.8)_ = 14.53, *p* < 0.001
**(G)** Cue × target × group		*F*_(3.3,157.1)_ = 0.9, *p* = 0.46	*F*_(3.7,177.8)_ = 1.44, *p* = 0.22
**Magnitude group differences**			
**(H)** Alerting	ANOVA	*F*_(2,95)_ = 2.63, *p* = 0.08	*F*_(2,95)_ = 4.85, *p* = 0.01
	HC-AD		(−0.010, 0.060), *p* = 0.25
*Post hoc*	HC-LBD		(0.009, 0.074), *p* = 0.008
	AD-LBD		(−0.013, 0.046), *p* = 0.52
**(I)** Orienting	ANOVA	*F*_(2,95)_ = 0.27, *p* = 0.77	*F*_(2,95)_ = 3.06, *p* = 0.052
**(J)** Executive	ANOVA	*F*_(2,95)_ = 5.30, *p* = 0.007	*F*_(2,95)_ = 3.01, *p* = 0.054
	HC-AD	(−325.5, −17.55), *p* = 0.02	(−0.111, 0.049), *p* = 1.0
*Post hoc*	HC-LBD	(−326.4, −39.1), *p* = 0.008	(−0.039, 0.111), *p* = 0.73
	AD-LBD	(−140.2, 117.7), *p* = 1.0	(−0.0001, 0.134), *p* = 0.05

*Repeated measures (cue × target) ANOVA effects with group (HC, AD, LBD) as between-subject factor (*F*-value, degrees of freedom (df), error df and *p*-value), and *Post hoc* tests (95% confidence interval of the mean difference, Bonferroni-corrected *p*-value). AD, Alzheimer’s disease; HC, healthy controls; LBD, Lewy body dementia; RT, reaction time*.

#### Alerting and Orienting Effects

There was a main effect of cue; across all groups mean RT for neutral cue trials was faster than no cue trials for both raw and normalized RT, i.e., there was an overall significant alerting effect. Furthermore, there was an overall significant orienting effect (difference between spatial and neutral cue trials) for raw and normalized RT (Table 3B).

When considering raw RT, the group × cue interaction was not significant, however it was significant for normalized RT. Subsequent *post hoc* tests at the individual group level for normalized RT revealed that controls had significant alerting and orienting effects. In AD and LBD, the alerting effect was not significant whereas both dementia groups demonstrated a significant orienting effect (Table 3D). The analysis of error rates did not show any difference between the different cue conditions with no significant cue × group interaction (Supplementary Tables S4B,D).

Comparing the magnitude of the alerting effect between groups did not reveal a significant difference for raw RT, whereas there was a significantly smaller alerting effect in LBD compared to HC when considering normalized RT (Table 3H). The orienting effect was not different in magnitude between groups for raw and normalized RT (Table 3I). Additionally, there were no group differences in the magnitude of the alerting and orienting effects for error rates (Supplementary Tables S5A,B).

There were no significant correlations between the alerting and orienting effects and any clinical variables after correcting for multiple comparisons (Supplementary Table S6).

#### Executive Conflict Effect

There was a main effect of target; across all groups mean RT for congruent trials was faster than for incongruent trials (Table 3C). Additionally, there was a significant target × group interaction (albeit only marginally significant for normalized RT) and *post hoc* tests revealed that the executive conflict effect was significant in all three groups (Table 3E). Comparing the magnitude of the effect between groups showed a larger conflict effect in both dementia groups compared to controls, with no difference between AD and LBD for raw RT. In contrast, for normalized RT there was no significant difference in the executive conflict effect between HC and either dementia group although there was a trend for a larger effect in AD than in LBD (Table 3J). Considering error rates, the executive conflict effect was significant in all groups (Supplementary Table S4C) and it was significantly larger in both AD and LBD compared to controls (Supplementary Tables S4E, S5C). There were no significant correlations between the executive conflict effect and clinical variables after correcting for multiple comparisons (Supplementary Table S6).

While there was a significant interaction between cue and target for both raw and normalized RT (Table 3F) and error rates (Supplementary Table S4F), these interactions did not differ between the groups (Table 3G and Supplementary Table S4G) and were therefore not analyzed further.

### Magnetic Resonance Imaging Analysis

Four DLB and two AD patients did not have structural scans and were therefore excluded from the VBM analysis.

**Figure 4:**
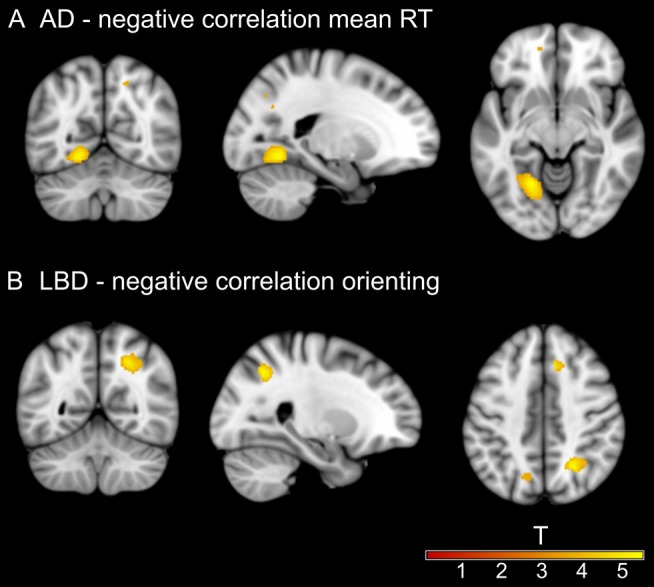
Significant clusters from voxel-based morphometry (VBM) analysis. **(A)** Negative correlation between mean RT and gray matter (GM) volume at left lingual gyrus in the AD group. **(B)** Negative correlation between the size of the orienting effect and white matter (WM) volume at right lateral occipital cortex in the LBD group. Information on all uncorrected results can be found in Supplementary Tables S7–S10.

In AD, there was a significant negative correlation between mean RT and GM volume in a large cluster at the lingual gyrus (Figure 4A). All other results in the AD group did not survive correction for multiple comparisons and are therefore reported as an exploratory analysis at a voxel-level threshold of *p* < 0.001 in Supplementary Tables S7, S9. The alerting effect negatively correlated with GM volume in parietal regions and positively correlated with WM in occipital regions. For the orienting effect there were positive correlations with GM volume in occipital and WM volume in temporal regions. Additionally, GM volume in different parts of the cerebellum in AD correlated with the executive conflict effect.

In LBD, there was a significant negative correlation between WM volume in a large cluster at the lateral occipital cortex and the orienting effect for raw and normalized RT (Figure 4B). Other results did not survive correction for multiple comparisons and are therefore reported at *p* < 0.001 in Supplementary Tables S8, S10. There were positive correlations between GM volume in temporal and parietal regions and the alerting effect. The orienting effect was positively correlated with GM volume in the parietal lobe and the executive conflict effect was negatively correlated with small GM clusters in temporal regions.

## Discussion

In this study, we investigated the efficiency of different attentional systems in AD and LBD compared to HC and the relationship between attentional deficits and brain structural abnormalities in the dementia groups. Both dementia groups showed slower overall mean RTs than controls, with additional slowing in the LBD group compared to AD. There was a significant alerting effect in controls which was absent in both dementia groups and the executive conflict effect was greater in the dementia groups compared to controls, while there were no group differences for the orienting effect. In the AD group mean RT was negatively correlated with occipital GM volume and orienting efficiency was negatively related to occipital WM volume in LBD.

### Mean Reaction Time

The overall RT slowing in both dementia groups, with additional slowing in LBD compared to AD, is replicating previous ANT studies in dementia cohorts (Fernandez-Duque and Black, 2006; Fuentes et al., 2010) and more general RT studies comparing AD and LBD (Ballard et al., 2001; Bradshaw et al., 2004). In AD, the observed RT slowing was related to diminished overall cognitive functioning, i.e., cognitively more impaired patients demonstrated more impaired RT performance. This result in AD is in agreement with a previous study which reported a slower RT during a choice RT task to be associated with reduced global cognitive functioning in AD and DLB (Ballard et al., 2001). In contrast to this previous study, we did not observe this relationship in our LBD group. An explanation for this lack of association may be that the DLB patients in Ballard et al. (2001) were cognitively more impaired than our LBD patients.

We also found a significant correlation between mean RT and GM volume at the lingual gyrus in AD, suggesting that lower brain volume in this area was related to general RT slowing. The ANT involves visual processing and thus RT slowing might be related to occipital/visual structural abnormalities in AD which agrees with previous studies showing associations between occipital cortex and visual impairments in AD and LBD (Firbank et al., 2003; Taylor et al., 2012; Ossenkoppele et al., 2015).

The accuracy with which participants performed the task was lower in both dementia groups compared to controls; however, error rates in LBD did not exceed those seen in AD indicating that LBD-related deficits seemed to be specific to response speed, with limited impact on accuracy.

### Alerting Effect

With respect to the alerting effect, group differences only became apparent when studying normalized RT which indicates that differences in alerting might have been obscured by overall differences in RT speed. The absence of a significant alerting effect in both dementia groups is indicative of reduced efficiency of the alerting system which seems to be a general dementia phenomenon and not specific to LBD or AD. In LBD, this result is expected given the association between alerting efficiency and noradrenergic projections from the locus coeruleus which are affected by LBD (Coull et al., 2001; Raz, 2004). In AD, however, this result stands in contrast to a previous study which found a significant alerting effect in this group (Fernandez-Duque and Black, 2006). AD patients in our study were cognitively more impaired compared to the AD patients in the previous study, which indicates that there might be a loss of alerting efficiency with dementia progression. The absence of a significant correlation between the size of the alerting effect and the severity of cognitive impairment in the AD group, however, indicates that this is not a linear relationship. Another explanation for the lack of a significant alerting effect in our study might be the modified ANT version that was used here. While Fernandez-Duque and Black (2006) used a display consisting of only two boxes that both lit up in the case of a neutral cue, in our version the neutral cue was conveyed by flashing the central box while the target was presented in one of the boxes either below or above the central box. Thus, participants were required to reorient their attention following a neutral cue and this may have had a detrimental effect on some participants’ RT performance, as indicated by negative alerting effects in some dementia patients (see Figure 2).

We did not find any significant associations between the alerting effect and gray and WM volume in either dementia group. An fMRI study of the efficiency of the different attentional components, using participants from the same study cohort as reported here, found LBD, AD and HC groups to have comparable fronto-parietal-occipital activations associated with the alerting effect (Firbank et al., 2016). Together with our results this suggests that the lack of a behavioral alerting effect in the dementia groups is unlikely to be due to region-specific functional or structural deficits.

### Orienting Effect

Regarding the orienting effect there were no group differences. This is comparable to previous literature showing preservation of the orienting system in AD patients (Fernandez-Duque and Black, 2006). However, given that the orienting system is postulated to be modulated by the basal forebrain cholinergic system (Fan et al., 2005), which is markedly affected in DLB (Clerici et al., 2007; Grothe et al., 2014), we expected the LBD group to exhibit reduced orienting efficiency. Given the marginally significant overall ANOVA for normalized RT and our prior hypothesis of reduced orienting efficiency in LBD, we conducted an exploratory *post hoc* analysis which demonstrated a marginally significant lower orienting effect in LBD compared to controls (*p* = 0.049), thus tentatively supporting our *a priori* hypothesis that this component of attention would be reduced in LBD, particularly when overall processing speed is considered. Orienting efficiency was related to WM abnormalities in occipital cortex in LBD indicating a relation between less efficient use of the orienting cue and reduced WM volume in lateral occipital cortex. Previous functional MRI studies have found brain activations for the orienting effect in occipital and parietal cortices (Fan et al., 2005; Firbank et al., 2016). Our results further suggest that structural alterations in these regions in LBD might also contribute to orienting inefficiency. Associations with occipital cortical volume were also found for alerting and orienting effects in AD. Even though these clusters did not survive multiple comparison correction they nevertheless suggest a trend for involvement of occipital regions in the efficiency of the ANT effects in both dementia groups.

### Executive Conflict Effect

The magnitude of the executive conflict effect was substantially greater in both dementia groups relative to controls, this is indicative of dementia patients exhibiting reduced ability to resolve conflict amongst responses which has previously been shown in AD (Fernandez-Duque and Black, 2006). Executive dysfunction is characteristic of LBD patients (Noe et al., 2004). Given that the dopaminergic system is postulated to have a regulatory role on the executive conflict component (Fan et al., 2005), the reduced executive conflict efficiency in the LBD patients fits with the notion of dopaminergic mediated frontal-striatal dysfunction being a contributory factor to the executive dysfunction in LBD (Kehagia et al., 2012). However, the group differences were greatly diminished when considering normalized RT, indicating that this effect was partly due to differences in overall processing speed. We did not see any strong relation between the efficiency of the executive conflict component and cortical volume in either dementia group. In particular, contrary to a previous study in individuals with MCI (Borsa et al., 2016), we did not see an association between GM volume at the ACC and the executive conflict effect in AD (even when considering uncorrected results). This might be due to higher inter-subject variability in ACC volume in MCI compared to AD. Furthermore, Borsa et al. (2016) restricted their analysis to relations between the ACC and the executive conflict effect which makes their analysis approach more sensitive compared to our whole-brain approach across all attentional components. Overall, our results for the executive conflict effect suggest that the dementia-related inefficiency of this component might be related to functional or microstructural rather than macrostructural changes.

### Clinical Correlations

Contrary to our hypothesis, we did not see any significant correlations between cognitive fluctuation severity and attentional measures in LBD. At an uncorrected threshold of *p* < 0.05, there was a negative correlation between the size of the alerting effect and the Mayo cognitive fluctuation score which indicates that there might be a relation between loss of alerting efficiency and more severe cognitive fluctuations in LBD. This would be in-line with previous reports in DLB that showed associations between greater RT slowing and cognitive fluctuations (Walker et al., 2000; Bradshaw et al., 2006). However, due to their exploratory nature our results should be interpreted with caution.

### Limitations

Our study has some limitations. Most patients were taking cholinergic and/or dopaminergic medication which is a potential confound as it is postulated that the orienting and executive conflict components are modulated by cholinergic and dopaminergic systems, respectively (Fan et al., 2005). Regarding dopaminergic medication, we showed that covarying for levodopa equivalent daily dose did not change the results (see “Effect of Dopaminergic Medication in the LBD Group” section of the Supplementary Material). However, the small number of patients who were not taking cholinesterase inhibitors did not allow for a more in-depth analysis of the effect of cholinergic medication on our results and therefore remains a limitation of this work. Furthermore, all diagnoses were based on clinical assessment rather than pathological confirmation. However, the standardized diagnostic criteria that were used in this study have demonstrated high specificity when validated against autopsy findings (McKeith et al., 2000). A further limitation might be that the two dementia groups were not completely matched in terms of overall cognitive impairment which might have influenced group comparisons. However, we repeated the analysis using data from two subgroups of AD and LBD patients that were matched in terms of overall cognition as measured by the MMSE and show that results remained largely the same (see “Analysis of Matched Dementia Subgroups” section of the Supplementary Material).

## Conclusion

To conclude, in contrast to previous studies in less impaired patients, we did not find a significant alerting effect for both dementia groups which might indicate that there is a loss of alerting efficiency with dementia progression. In contrast, orienting was largely preserved with a slight impairment in LBD that was not observed in AD and this might be related to structural abnormalities in occipital regions. Finally, the resolution of executive conflict was clearly impaired in both dementia groups but did not appear to be related to macrostructural changes in brain volume in either group.

## Author Contributions

RC and JS: study conception, data analysis and interpretation, drafting and revision of the manuscript. RC, AK and MF: data acquisition. SG, PG, LP, MF and J-PT: study conception, data interpretation and revision of the manuscript. AB, MK, AT and JO’B: study conception and revision of the manuscript.

## Conflict of Interest Statement

The authors declare that the research was conducted in the absence of any commercial or financial relationships that could be construed as a potential conflict of interest.

## Footnotes

^1^http://www.vislab.ucl.ac.uk/cogent_2000.php

^2^www.fil.ion.ucl.ac.uk/spm/

^3^www.restfmri.net
