# Supplementary material for: Structural Brain Correlates of Attention Dysfunction in Lewy Body Dementias and Alzheimer’s Disease
Source: Front Aging Neurosci. 2018 Oct 30;10:347. doi: 10.3389/fnagi.2018.00347 (PMC6251343; doi:10.3389/fnagi.2018.00347)
Supplement: Supplementary file 1 [file Data_Sheet_1.pdf]

Supplementary Material

Structural brain correlates of attention dysfunction in Lewy body dementias and Alzheimer's disease

Ruth Cromarty[1†], Julia Schumacher[1†*], Sara Graziadio[2], Peter Gallagher[3], Alison Killen[1], Michael J. Firbank[1], Andrew Blamire[4], Marcus Kaiser[1,5], Alan J. Thomas[1], John T. O'Brien[6], Luis R. Peraza[1,5] and John-Paul Taylor[1]

*** Correspondence:** Julia Schumacher, j.a.schumacher2@newcastle.ac.uk

Contents

1 Comparison of DLB and PDD subgroups 2
2 Analysis of error rates 4
3 Clinical correlations in AD and LBD 6
4 Results from VBM analysis 7
5 Effect of dopaminergic medication in the LBD group 12
6 Analysis of matched dementia subgroups 13
7 Supplementary references 15

1 Comparison of DLB and PDD subgroups

Supplementary Table S1: Demographic and clinical comparison of DLB and PDD patients.

	DLB (N=25)	PDD (N=20)	Between-group differences
Male: female	19:6	19:1	χ^2=3.05, p=0.08[a]
Age	76.1 (6.2)	72.6 (5.9)	t_{43}=1.95, p=0.06[c]
AChEI	23	16	χ^2 =1.39, p =0.24[a]
PD meds	13	20	χ^2 =13.09, p<0.001[a]
Duration	3.6 (2.4)	2.6 (1.5)	U=1.14, p=0.29[b]
MMSE	23.0 (4.2)	23.7 (3.2)	t_{43}=0.54, p =0.60[c]
CAMCOG	74.5 (14.7)	77.7 (9.3)	t_{43}=0.85, p =0.40[c]
UPDRS	15.6 (7.2)	26.6 (7.9)	t_{43}=4.86, p<0.001[c]
CAF total	4.00 (4.4)	6.6 (4.3)[d]	t_{41}=1.89, p=0.07[c]
Mayo total	12.3 (6.2)	15.2 (5.0)[d]	t_{41}=1.67, p=0.10[c]
Mayo cogn	2.2 (1.8)	3.7 (1.8)[d]	t_{41}=2.59, p=0.01[c]
NPI total	9.1 (4.9)	18.8 (11.6)	t_{43}=3.78, p<0.001[c]
NPI hall	1.4 (1.7)	2.2 (2.2)	t_{43}=1.37, p=0.18[c]

AChEI, number of patients taking acetylcholinesterase inhibitors; CAF total, Clinical Assessment of Fluctuations total score; CAMCOG, Cambridge Cognitive Examination; DLB, Dementia with Lewy bodies; Duration, duration of cognitive symptoms in years; Mayo total, Mayo Fluctuations Scale; Mayo cognitive, Mayo Fluctuation cognitive subscale; Mayo arousal, Mayo Fluctuations arousal subscale; MMSE, Mini Mental State Examination; PDD, Parkinson's disease dementia; PD meds, number of patients taking dopaminergic medication; UPDRS, Unified Parkinson's Disease Rating Scale; NPI, Neuropsychiatric Inventory; NPI hall, NPI hallucination subscore

[a] Chi-square test DLB, PDD; [b] Mann Whitney U test DLB, PDD; [c] Student's t-test DLB, PDD; [d]N=18.

Supplementary Table S2: Mean reaction times, error rates, and ANT effects for DLB and PDD subgroups (standard deviations are presented in brackets). Comparison between groups using independent samples t-tests.

	DLB (N=25)	PDD (N=20)	Between-group differences
Mean RT	1483.6 (382.3)	1651.7 (391.0)	t_{43}=1.45, p=0.15
Mean error rate (%)	14.2 (8.4)	12.2 (10.0)	t_{43}=0.74, p=0.47
Alerting			
Raw RT	10.9 (87.7)	-11.2 (101.7)	t_{43}=0.78, p=0.44
Normalised RT	0.01 (0.06)	-0.0006 (0.05)	t_{43}=0.65, p=0.52
Orienting			
Raw RT	85.0 (98.8)	82.5 (131.1)	t_{43}=0.07, p=0.94
Normalised RT	0.06 (0.06)	0.05 (0.07)	t_{43}=0.19, p=0.85
Executive			
Raw RT	548.9 (281.6)	596.7 (219.4)	t_{43}=0.62, p=0.54
Normalised RT	0.35 (0.12)	0.36 (0.10)	t_{43}=0.32, p=0.75

DLB, dementia with Lewy bodies; PDD, Parkinson's disease dementia; RT, reaction time

2 Analysis of error rates

Supplementary Table S3: Mean error rates (%) for each task condition (cue x target), for the controls, AD and LBD patients. Standard deviations are presented in brackets

		HC (n=22)	AD (n=31)	LBD (n=45)
Mean error rates (%)				
All trials		1.8 (1.7)	9.7 (8.6)	13.3 (9.1)
	Overall	1.9 (2.4)	9.8 (7.5)	14.1 (10.3)
No Cue	Congruent	1.1 (2.1)	4.3 (6.0)	5.8 (6.0)
	Incongruent	2.8 (3.8)	15.3 (11.6)	22.4 (17.5)
	Overall	2.1 (1.8)	9.5 (9.1)	12.8 (9.1)
Neutral	Congruent	1.1 (1.5)	5.4 (5.8)	7.6 (8.1)
	Incongruent	3.1 (2.6)	13.7 (13.4)	18.0 (14.2)
	Overall	1.5 (1.8)	9.8 (9.9)	13.0 (9.5)
Spatial	Congruent	0.7 (1.7)	5.2 (7.6)	6.4 (7.5)
	Incongruent	2.3 (2.5)	14.5 (15.0)	19.6 (14.2)
Congruent	Overall	0.9 (1.5)	4.9 (6.0)	6.6 (6.2)
Incongruent	Overall	2.7 (2.4)	14.5 (12.8)	20.0 (14.3)
ANT effects (%)				
Alerting		-0.2 (2.2)	0.2 (3.5)	1.3 (3.5)
Orienting		0.6 (1.3)	-0.3 (3.7)	-0.3 (3.7)
Executive		1.78 (2.1)*	9.7 (10.2)*	9.6 (10.2)*

AD, Alzheimer’s disease; HC, healthy controls; LBD, Lewy body dementia;
*Significant ANT effect, p-value < 0.05 for error rates

Supplementary Table S4: Results from statistical tests for error rates. Repeated measures (cue x target) ANOVA effects with group (HC, AD, LBD) as between-subject factor (F value, degrees of freedom (df), error df, and p-value), and post-hoc tests (95% confidence interval, Bonferroni-corrected p-value).

		Effect significance, error rates
Main effects		
A) Group		F(2,95)=15.59, p<0.001
	HC-AD	[-13.3, -2.5], p=0.002
Post-hoc	HC-LBD	[-16.5, -6.5], p<0.001
	AD-LBD	[-8.1, 0.9], p=0.17
B) Cue		F(1.8,172.4)=0.67, p=0.51
C) Target		F(1,95)=57.70, p<0.001
D) Cue x group		F(3.6,172.4)=0.65, p=0.63
Interactions		
E) Target x group		F(2,95)=9.38, p<0.001
HC	Executive	F(1,21)=15.98, p=0.001
AD	Executive	F(1,30)=27.10, p<0.001
LBD	Executive	F(1,44)=51.82, p<0.001
F) Cue x target		F(1.9,176.2)=3.87, p=0.02
G) Cue x target x group		F(3.7,176.2)=1.76, p=0.14

AD, Alzheimer’s disease; HC, healthy controls; LBD, Lewy body dementia

Supplementary Table S5: Comparison of magnitude of ANT effects between the groups for error rates using univariate ANOVAs with ANT effect as dependent variable and group as fixed factor (F value, degrees of freedom (df), error df, and p-value) and post-hoc tests (95% confidence interval, Bonferroni-corrected p-value).

		Effect significance, error rates
A) alerting		
ANOVA		F(2,95)=0.79, p=0.46
B) orienting		
ANOVA		F(2,95)=0.44, p=0.65
C) executive		
ANOVA		F(2,95)=9.38, p<0.001
	HC-AD	[-14.8, -0.8], p=0.02
Post-hoc	HC-LBD	[-18.1, -5.1], p<0.001
	AD-LBD	[-9.6, 2.0], p=0.35

AD, Alzheimer's disease; HC, healthy controls; LBD, Lewy body dementia

3 Clinical correlations in AD and LBD

Supplementary Table S6: Pearson's correlations between clinical scores and ANT effects using normalized and raw RT in the dementia groups. Correlation value (uncorrected p-value, p-value FDR-corrected for multiple comparisons). Correlations surviving FDR-correction are marked with an asterisk.

		Raw reaction time	Normalized reaction time
AD			
Mean RT	MMSE	-0.54 (0.002, 0.04)*	/
	CAMCOG	-0.54 (0.002, 0.04)*	/
Alerting	CAMCOG	-0.38 (0.036, 0.26)	-0.23 (0.20, 0.60)
Orienting	CAMCOG	0.36 (0.046, 0.26)	0.43 (0.02, 0.32)
LBD			
Mean RT	UPDRS	0.39 (0.008, 0.13)	/
	Mayo cogn[a]	0.33 (0.03, 0.26)	/
Alerting	MMSE	0.29 (0.055, 0.26)	0.31 (0.04, 0.32)
	Mayo total	-0.28 (0.07, 0.26)	-0.32 (0.03, 0.32)
	Mayo cogn[a]	-0.33 (0.03, 0.26)	-0.35 (0.02, 0.32)

AD, Alzheimer's disease; CAMCOG, Cambridge Cognitive Examination; LBD, Lewy body dementia; Mayo cogn, Mayo Fluctuations cognitive subscale; Mayo total, Mayo Fluctuations Scale; MMSE, Mini Mental State Examination; NPI hall, Neuropsychiatric Inventory hallucination subscore; RT, reaction time; UPDRS, Unified Parkinson's Disease Rating Scale
[a]N=43

4 Results from VBM analysis

Supplementary Table S7: Correlations between ANT effects (normalized RT) and grey matter (GM) and white matter (WM) volume in AD. All clusters are significant at $p<0.001$, uncorrected. Correction for multiple comparisons was performed using Monte-Carlo simulations with AlphaSim at $p<0.05$ resulting in minimum cluster sizes of 257 (GM) and 258 (WM) voxels. No cluster survived correction. Locations were estimated from the Harvard-Oxford atlas in FSL and WM regions were identified from the nearest GM structure.

Grey matter			White matter		
Cluster location	size	MNI (X,Y,Z)	Cluster location	size	MNI (X,Y,Z)
Alerting, negative correlation					
R postcentral gyrus	127	66,-10,15	No significant clusters		
L anterior supramarginal	101	-52,-27,38			
L anterior supramarginal	41	-60,-34,45			
R posterior supramarginal	8	68,-42,15			
R frontal orbital cortex	2	24,30,-26			
Alerting, positive correlation					
No significant clusters			L lateral occipital cortex	67	-38,-64,34
			R lateral occipital cortex	14	44,-63,9
			R lateral occipital cortex	2	33,-86,9
			R angular gyrus	2	44,-56,30
			L temporal fusiform	1	-39,-32,-18
			L precuneus	1	-10,-54,60
Orienting, negative correlation					
No significant clusters			No significant clusters		
Orienting, positive correlation					
R posterior middle temporal	74	68,-26,-12	R inferior temporal gyrus	95	56,-39,-18
R occipital pole	33	33,-92,20	R temporal occipital	10	34,-56,0
R superior lateral occipital	3	21,-60,64	L middle temporal gyrus	8	-54,-34,-10
R superior lateral occipital	2	26,-68,50	R lateral occipital cortex	5	38,-68,32
			R middle temporal gyrus	3	36,-58,15
			L middle frontal gyrus	1	-39,22,39
Executive, negative correlation					
R cerebellum Crus I	79	48,-74,-30	No significant clusters		
R paracingulate gyrus	34	6,40,36			
Executive, positive correlation					
R cerebellum V	22	9,-58,-27	R precuneus	2	16,-64,40
L cerebellum VIIb	1	-27,-66,-44			

Supplementary Table S8: Correlations between ANT effects (normalized RT) and grey matter (GM) and white matter (WM) volume in LBD. All clusters are significant at p<0.001, uncorrected. Correction for multiple comparisons was performed using Monte-Carlo simulations with AlphaSim at p<0.05 resulting in minimum cluster sizes 220 (GM) and 260 (WM) voxels. Clusters surviving multiple comparison correction are highlighted with an asterisk. Locations were estimated from the Harvard-Oxford atlas in FSL and WM regions were identified from the nearest GM structure.

Grey matter			White matter		
Cluster location	size	MNI (X,Y,Z)	Cluster location	size	MNI (X,Y,Z)
Alerting, negative correlation					
L lateral occipital cortex	22	-20,-75,45	No significant clusters		
Alerting, positive correlation					
R temporal pole	79	18,3,-42	No significant clusters		
R temporal fusiform	16	39,-15,-26			
L frontal pole	7	-9,44,52			
R temporal pole	1	22,16,-36			
Orienting, negative correlation					
No significant clusters			R lateral occipital cortex	325*	24,-58,45
			L frontal pole	34	-33,39,20
			R supplementary motor area	10	8,-10,52
			R inferior temporal gyrus	8	45,-28,-22
			L precentral gyrus	8	-8,-14,51
			R paracingulate gyrus	6	9,24,44
			R angular gyrus	2	50,-46,18
Orienting, positive correlation					
R angular gyrus	126	45,-45,20	R occipital pole	4	24,-93,15
L parahippocampal gyrus	40	-12,-38,-6			
L precentral gyrus	38	-57,9,2			
R frontal pole	26	28,46,34			
R angular gyrus	19	62,-52,38			
L insular cortex	14	-44,-4,10			
L frontal pole	7	-30,36,-20			
L postcentral gyrus	7	-40,-33,50			
R middle frontal gyrus	6	32,30,30			
R frontal pole	3	3,64,6			
R inferior frontal gyrus	2	52,20,30			
R supramarginal gyrus	1	50,-30,52			
Executive, negative correlation					
R temporal pole	42	39,14,-48	No significant clusters		
L frontal pole	34	-51,42,-6			
Executive, positive correlation					
R parahippocampal gyrus	5	15,2,-27	No significant clusters		
R temporal pole	2	21,10,-46			

Supplementary Table S9: Correlations between mean RT and ANT effects (raw RT) and grey matter (GM) and white matter (WM) volume in AD. All clusters are significant at p<0.001, uncorrected. Correction for multiple comparisons was performed using Monte-Carlo simulations with AlphaSim at p<0.05 resulting in minimum cluster sizes of 223 (GM) and 233 (WM) voxels for mean RT and 256 (GM) and 228 (WM) voxels for ANT effects. Clusters surviving multiple comparison correction are highlighted with an asterisk. Locations were estimated from the Harvard-Oxford atlas in FSL and WM regions were identified from the nearest GM structure.

Grey matter			White matter		
Cluster location	size	MNI (X,Y,Z)	Cluster location	size	MNI (X,Y,Z)
Mean RT, negative correlation					
L lingual gyrus	805*	-21,-58,-9	R inferior frontal gyrus	193	51,33,8
L angular gyrus	74	-45,-50,22	L postcentral gyrus	92	-54,-14,22
L paracingulate gyrus	56	-12,52,-6	L middle temporal gyrus	85	-56,-36,-14
R cerebellum Crus I	43	20,-86,-22	L lateral occipital cortex	49	-33,-69,0
L middle frontal gyrus	31	-34,14,36	L occipital fusiform gyrus	41	-22,-66,-8
L posterior supramarginal	30	-58,-46,30	R lateral occipital cortex	38	27,-82,21
L superior lateral occipital	14	18,-62,50	L lingual gyrus	8	-12,-72,-8
L cerebellum Crus I	13	-24,-87,-28	L precentral gyrus	8	-46,-9,28
R occipital pole	9	36,-93,9	R inferior temporal gyrus	3	57,-36,-21
L superior lateral occipital	7	-18,-69,40	R frontal pole	2	45,40,-8
L precuneus	3	-20,-64,30			
L precuneus	3	-12,-69,28			
L temporal occipital fusiform	2	-46,-58,-20			
Mean RT, positive correlation					
No significant clusters			No significant clusters		
Alerting, negative correlation					
L anterior supramarginal	129	-51,-27,36	No significant clusters		
L posterior cingulate	26	-9,-22,44			
R poscentral gyrus	20	66,-10,14			
L anterior supramarginal	14	-60,-34,45			
L frontal orbital cortex	11	-22,32,-26			
R occipital pole	9	26,-87,32			
R inferior frontal gyrus	3	56,28,20			
L superior parietal lobule	3	-27,-40,51			
Alerting, positive correlation					
No significant clusters			L lateral occipital cortex	36	-38,-64,33
Orienting, negative correlation					
R frontal pole	7	52,39,15	No significant clusters		
Orienting, positive correlation					
R occipital pole	74	33,-92,20	R inferior temporal gyrus	7	56,-39,-18
R lateral occipital cortex	47	34,-76,39			
L superior lateral occipital	23	-27,-80,32			
R frontal pole	19	34,57,22			
R superior lateral occipital	16	34,-72,20			
R superior lateral occipital	15	26,-68,50			

R posterior middle temporal	3	69,-26,-12			
R lingual gyrus	2	20,-63,-9			
Executive, negative correlation					
R paracingulate gyrus	22	6,40,34	L central opercular	1	-51,-15,18
R cerebellum Crus I	20	48,-74,-30			
R cerebellum Crus I	11	34,-82,-21			
R frontal pole	1	34,40,38			
Executive, positive correlation					
R cerebellum V	24	8,-57,-27	L cerebellum VIIIa	1	-28,-50,-44
R cerebellum Crus II	19	32,-63,-42			

Supplementary Table S10: Correlations between mean RT and ANT effects (raw RT) and grey matter (GM) and white matter (WM) volume in LBD. All clusters are significant at p<0.001, uncorrected. Correction for multiple comparisons was performed using Monte-Carlo simulations with AlphaSim at p<0.05 resulting in minimum cluster sizes of 230 (GM) and 257 (WM) voxels for mean RT and 242 (GM) and 262 (WM) voxels for ANT effects. Clusters surviving multiple comparison correction are highlighted with an asterisk. Locations were estimated from the Harvard-Oxford atlas in FSL and WM regions were identified from the nearest GM structure.

Grey matter			White matter		
Cluster location	size	MNI (X,Y,Z)	Cluster location	size	MNI (X,Y,Z)
Mean RT, negative correlation					
No significant clusters			No significant clusters		
Mean RT, positive correlation					
L frontal pole	79	-24,58,27	R cerebellum I-IV	6	9,-48,-21
L superior parietal lobule	23	-44,-40,54	R temporal fusiform	2	39,-16,-22
L cerebellum X	7	-24,-40,-44	R temporal fusiform	1	39,-21,-22
R cerebellum V	1	4,-56,-12			
Alerting, negative correlation					
L postcentral gyrus	2	-18,-34,76	L inferior temporal	2	-54,-48,-16
L lingual gyrus	1	-20,-75,-3			
Alerting, positive correlation					
R parahippocampal gyrus	120	18,3,-42	No significant clusters		
L frontal pole	21	-8,44,51			
R posterior temporal fusiform	11	39,-15,-27			
R posterior cingulate	4	9,-36,45			
Orienting, negative correlation					
No significant clusters			R lateral occipital cortex	484*	24,-58,45
			R paracingulate gyrus	159	9,22,45
			R supplementary motor area	80	8,-10,54
			L supplementary motor area	80	-8,-12,54
			L frontal pole	79	-33,39,18
			L precuneus	74	-8,-64,46
			R angular gyrus	21	50,-46,18
			R inferior temporal gyrus	17	45,-28,-22
			R paracingulate gyrus	9	9,34,38
			R precuneus	2	22,-60,24
			R middle temporal gyrus	2	54,-52,-6
			L supramarginal gyrus	1	-40,-40,39
			L posterior cingulate	1	-9,-42,42
Orienting, positive correlation					
L parahippocampal gyrus	81	-12,-38,-6	R occipital pole	12	24,-93,15
R frontal pole	11	30,48,34			
R angular gyrus	11	46,-50,27			
R frontal pole	5	3,64,6			
L postcentral gyrus	1	-42,-34,51			

Executive, negative correlation					
R temporal pole	38	39,15,-46	No significant clusters		
Executive, positive correlation					
R frontal pole	7	42,54,18	R precuneus	1	12,-52,62
R cerebellum VIIIb	2	12,-60,-39			
L frontal pole	1	-26,57,27			

5 Effect of dopaminergic medication in the LBD group

To study possible effects of dopaminergic medication on ANT effects in the LBD group, the repeated-measures (cue x target) ANOVA was repeated including a covariate for daily levodopa equivalent dose (LED, Tomlinson et al., 2010). This was tested for both raw and normalised RT.
For raw RT, there was no interaction between LED and cue ($F(2,84)=0.15$, $p=0.86$) or target ($F(1,42)=0.003$, $p=0.96$). There was a main effect of cue ($F(2,84)=9.34$, $p<0.001$) with post-hoc tests revealing no alerting effect (no cue compared to neutral cue, 95% confidence interval (CI)=[-34.4, 37.5], $p=1.0$), but a significant orienting effect (neutral cue compared to spatial cue, 95% CI=[39.7, 126.4], $p<0.001$). Furthermore, there was a main effect of target with slower RTs in incongruent compared to congruent trials ($F(1,42)=104.1$, $p<0.001$).
For normalised RT, there was no interaction between LED and cue ($F(2,84)=0.08$, $p=0.92$) and no target by LED interaction ($F(1,42)=0.85$, $p=0.36$). There was a main effect of cue ($F(2,84)=12.20$, $p<0.001$) with no alerting effect (95% CI=[-0.02, 0.03], $p=1.0$), but a significant orienting effect (95% CI=[0.03, 0.08], $p<0.001$). There was also a significant main effect of target ($F(1,42)=224.01$, $p<0.001$).
These results are comparable to the results without LED covariate, thus indicating that dopaminergic medication dose does not influence the ANT effects in LBD.

6 Analysis of matched dementia subgroups

To ensure that differences in overall cognitive impairment between AD and LBD did not influence the group comparisons, we repeated all statistical analyses for subgroups of AD and LBD patients that were matched in terms of MMSE. To create these groups, four AD patients with MMSE<16 and ten LBD patients (six DLB and four PDD) with MMSE>26 were excluded from the analysis. Supplementary Table S11 show demographic and clinical information about the dementia subgroups. Supplementary Table S12 shows results from the same statistical tests as in Table 2 of the main text when restricting the analysis to the matched subgroups.

Supplementary Table S11: Demographics and clinical information for matched dementia subgroups; mean (standard deviation)

	AD (n=27)	LBD (n=35)	Between-group differences
Male: female	21:6	29:6	χ^2=0.25, p=0.62[a]
Age	76.5 (7.9)	74.5 (6.7)	t_{60}=1.06, p=0.29[b]
AChEI	26	31	χ^2=1.23, p=0.27[a]
Dopaminergic medication	0	26	χ^2=34.54, p<0.001[a]
Duration	3.9 (2.2)	3.2 (2.2)	U=358, p=0.10[c]
MMSE	21.7 (2.8)	22.0 (3.2)	t_{60}=0.38, p=0.71[b]
CAMCOG	71.1 (9.9)	72.1 (11.5)	t_{60}=0.39, p=0.70[b]
UPDRS	2.3 (2.3)	21.4 (9.5)	t_{60}=10.2, p<0.001[b]
CAF total	0.8 (1.7)[d]	5.8 (4.6)[e]	t_{58}=5.4, p<0.001[b]
Mayo total	8.5 (4.0)[d]	15.3 (4.9)[e]	t_{58}=5.8, p<0.001[b]
Mayo cogn	1.6 (1.7)[d]	3.4 (1.7)[e]	t_{58}=3.9, p<0.001[b]
NPI total	7.2 (6.6)[d]	14.5 (9.9)	t_{59}=3.3, p=0.002[b]
NPI hall	0.04 (0.2)[d]	1.7 (1.8)	t_{59}=4.6, p<0.001[b]

AChEI, number of patients taking acetylcholinesterase inhibitors; AD, Alzheimer's disease; CAF total, Clinical Assessment of Fluctuations total score; CAMCOG, Cambridge Cognitive Examination; Duration, duration of cognitive symptoms in years; LBD, Lewy body dementia; Mayo Fluctuations, Mayo Fluctuations cognitive subscale; MMSE, Mini Mental State Examination; na, not applicable; Dopaminergic medication, number of patients taking dopaminergic medication; UPDRS, Unified Parkinson's Disease Rating Scale III; NPI, Neuropsychiatric Inventory; NPI hall, NPI hallucination subscore.

[a] Chi-square test AD, LBD; [b] Student's t-test AD, LBD; [c] Mann Whitney U test AD, LBD; [d]N=26, [e]N=35

Supplementary Table S12: Results from statistical tests for raw and normalized reaction times analyzing matched dementia subgroups. Repeated measures (cue x target) ANOVA effects with group (HC, AD, LBD) as between-subject factor (F value, degrees of freedom (df), error df, and p-value), and post-hoc tests (95% confidence interval of the mean difference, Bonferroni-corrected p-value).

		Effect significance, raw RT	Effect significance, normalized RT
Main effects			
A) Group		F(2,81)=29.6, p<0.001	
Post-hoc	HC-AD	[-557.0, -118.7], p=0.001	
	HC-LBD	[-857.5, -442.3], p<0.001	
	AD-LBD	[-507.4, -116.6], p=0.001	
B) Cue		F(2,162)=63.3, p<0.001	F(2,162)=115.9, p<0.001
Post-hoc	Alerting	[-4.2, 37.6], p=0.16	[0.006, 0.032], p=0.002
	Orienting	[59.9, 109.1], p<0.001	[0.073, 0.106], p<0.001
C) Target		F(1,81)=440.1, p<0.001	F(1,81)=978.0, p<0.001
Interactions			
D) Cue x group		F(4,162)=2.00, p=0.10	F(4,162)=8.4, p<0.001
HC	Cue		F(2,42)=167.0, p<0.001
	Alerting		[0.026, 0.068], p<0.001
	Orienting		[0.073, 0.111], p<0.001
AD	Cue		F(2,52)=27.3, p<0.001
	Alerting		[-0.012, 0.037], p=0.62
	Orienting		[0.044, 0.113], p<0.001
LBD	Cue		F(2,68)=16.4, p<0.001
	Alerting		[-0.025, 0.020], p=1.0
	Orienting		[0.024, 0.078], p<0.001
E) Target x group		F(2,81)=6.4, p=0.003	F(2,81)=3.10, p=0.051
HC	Executive	F(1,21)=111.68, p<0.001	F(1,21)=227.05, p<0.001
AD	Executive	F(1,26)=187.8, p<0.001	F(1,26)=428.5, p<0.001
LBD	Executive	F(1,34)=194.6, p<0.001	F(1,34)=361.2, p<0.001
F) Cue x target		F(1.6,132.9)=4.7, p=0.01	F(1.9,150.5)=10.7, p<0.001
G) Cue x target x group		F(3.3,132.9)=1.1, p=0.37	F(3.7,150.4)=1.39, p=0.24
Magnitude group differences			
H) alerting	ANOVA	F(2,81)=3.58, p=0.03	F(2,81)=7.0, p=0.002
Post-hoc	HC-AD	[-21.5,86.5], p=0.44	[0.000, 0.068], p=0.047
	HC-LBD	[4.8,107.1], p=0.03	[0.017, 0.081], p=0.001
	AD-LBD	[-24.7,71.7], p=0.71	[-0.016, 0.045], p=0.72
I) orienting	ANOVA	F(2,81)=0.15, p=0.87	F(2,81)=3.0, p=0.055
J) executive	ANOVA	F(2,81)=6.4, p=0.003	F(2,81)=3.1, p=0.051
Post-hoc	HC-AD	[-341.6, -29.7], p=0.01	[-0.120, 0.039], p=0.65
	HC-LBD	[-353.3, -57.7], p=0.003	[-0.044, 0.107], p=0.93
	AD-LBD	[-159.0, 119.3], p=1.0	[0.001, 0.143], p=0.05

AD, Alzheimer's disease; HC, healthy controls; LBD, Lewy body dementia; RT, reaction time

7 Supplementary references

Tomlinson, C. L., Stowe, R., Patel, S., Rick, C., Gray, R., and Clarke, C. E. (2010). Systematic review of levodopa dose equivalency reporting in Parkinson's disease. *Mov. Disord.* 25, 2649–2653. doi:10.1002/mds.23429.
